# Component resolved diagnosis in relation to severity of hazelnut allergy across Europe

**DOI:** 10.1186/2045-7022-3-S3-P39

**Published:** 2013-07-25

**Authors:** MR Datema, L Zuidmeer, C Garino, J Marsh, A Lovegrove, M Clausen, D Gislason, R Dubakiene, I Reig, L Barreales, AC Knulst, T-M Le, ML Kowalski, M Jedvzejczak, J Lidholm, T Kralimarkova, T Popov, R Asero, S Seneviratne, N Sinaniotis, N Papadopoulos, A Purohit, F de Blay, P Bures, S Vieths, M Fernández-Rivas, K Hoffmann-Sommergruber, R van Ree, B Ballmer-Weber

**Affiliations:** 1 Experimental Immunology, Academic Medical Center, Amsterdam, the Netherlands; 2 DiSCAFF & Drug and Food Biotechnological Center, Università del Piemonte Orientale, Novara, Italy; 3 Rothamsted Research, Harpenden, Hertfordshire, UK; 4 Landspitali University Hospital, Reykjavík, Iceland; 5 Vilnius University, Vilnius, Lithuania; 6 Allergy Department, Hospital Clínico San Carlos, Madrid, Spain; 7 Department of Dermatology/Allergology, University Medical Center Utrecht, Utrecht, the Netherlands; 8 Department of Immunology, Rheumatology and Allergy, Faculty of Medicine, Medical University of Lodz, Lodz, Poland; 9 Phadia AB, Uppsala, Sweden; 10 Clinical Centre of Allergology, Alexandrovska Hospital, Sofia, Bulgaria; 11 Ambulatorio di Allergologia, Clinica San Carlo, Paderno Dugnano, Italy; 12 Department of Respiratory Epidemiology and Public Health, National Heart and Lung Institute, Imperial College London, London, UK; 13 Allergy Department, 2nd Pediatric Clinic, University of Athens, Athens, Greece; 14 Division of Allergy, Department of Chest Diseases, University Hospitals of Strasbourg, Strasbourg, France; 15 Allergy Unit, Department of Dermatology, University Hospital of Zürich, Zürich, Switzerland; 16 Division of Allergology, Paul-Ehrlich-Institut, Langen, Germany; 17 Department of Pathophysiology, Medical University of Vienna, Vienna, Austria

## Background

Hazelnut is one of the most common food allergies across Europe although sensitization profiles to specific hazelnut allergens differ between countries. We aim explore geographical differences across Europe regarding molecular sensitization profiles and asses possible associations with severity of hazelnut allergy.

## Methods

This study was part of EuroPrevall, a multi-center European study on food allergies. A cohort of 2272 subjects from 12 European countries with reported allergy to at least one of 24 common foods was tested by ImmunoCAP (CAP) and skin prick test (SPT) to a panel of 24 foods, 12 inhalant allergens and latex. Subjects with a convincing history of hazelnut allergy (n=739) were included in this study. Component-resolved diagnosis (CRD) was performed on 426/739 with well-characterized purified recombinant (r) Cor a 1 (Bet v 1-homologue), rCor a 2 (profilin), rCor a 8 (LTP), rCor a 9 (legumin), rCor a 11 (vicilin), nCor a 12 (oleosin), rCor a 14 (2S albumin) and MUXF (CCD). Double-blind placebo-controlled (DBPCFC) food challenges were performed with 128 subjects.

## Results

Hazelnut ranked 1^st^ across Europe in an outpatient clinic population, with 739subjects (32.5 %) reporting a convincing clinical history, ranging from 26.4% to 68.6% in Czech Republic, Poland, the Netherlands, France, United Kingdom, Italy, Switzerland and Lithuania and 4.9-12.0% in Spain, Bulgaria, Iceland and Greece. Sensitization to Cor a 1 was most prevalent in countries with a high percentage of birch pollen allergy. Cor a 8 sensitization was mostly seen in Greek patients (83%) and to a lesser extent in Spain (36.6%). Sensitization other allergens was in general much lower although present in subgroups of the population; Cor a 9 sensitization was observed more often in younger patients and Cor a 8, Cor a 9, Cor a 11, Cor a 12 and Cor a 14 were more prevalent in atopic dermatitis patients. Studies into putative associations between hazelnut allergens and symptom severity are still ongoing and will also be stratified on geographical area.

## Conclusion

The outcome of this study so far confirms the results from previous studies. This standardized multinational study provides a unique set of data regarding hazelnut allergy and sensitization profiles.

## Disclosure of interest

None declared.

